# Regular Metronome, Fractal Metronome, and Music for Parkinson Gait

**DOI:** 10.1001/jamanetworkopen.2026.2744

**Published:** 2026-04-16

**Authors:** Kristen L. Sowalsky, Michael S. Okun, Matthew Terza, A. Enrique Martinez-Nunez, Hyokeun Lee, Nikolaus R. McFarland, Nikolaos Stergiou, Leonardo Almeida, Chris J. Hass

**Affiliations:** 1 Department of Applied Physiology and Kinesiology, University of Florida, Gainesville; 2 Department of Neurology, Norman Fixel Institute for Neurological Diseases, University of Florida, Gainesville; 3 Department of Sports Convergence Science, Kwangwoon University, Seoul, South Korea; 4 Department of Biomechanics, University of Nebraska Omaha, Omaha; 5 Department of Physical Education and Sport Science, Aristotle University of Thessaloniki, Thessaloniki, Greece; 6 Center for Research in Human Movement Variability, University of Nebraska Omaha, Omaha

## Abstract

**Importance:**

Persons with Parkinson walk slowly, with short steps, reduced arm swing, and altered stride variability. Walking to a metronome or music may increase velocity, stride length, and cadence, yet few studies have directly compared the efficacy of these techniques.

**Objective:**

To determine the optimal auditory cue to improve Parkinson gait.

**Design, Setting, and Participants:**

This case-control study was conducted among persons with Parkinson disease (using medication) and healthy older adult controls at University of Florida Applied Neuromechanics laboratory in 2017. Changes in gait in participants with Parkinson disease and controls were compared with walking with no cue: walking to a regular metronome, walking to a fractal metronome, and walking to music. Condition order was randomized. Cueing frequency was set to natural cadence. Post hoc analyses restricted to participants with Parkinson disease were performed in 2026.

**Exposures:**

Auditory cueing with a regular metronome, fractal metronome, or music while walking.

**Main Outcomes and Measures:**

Outcomes of interest were stride time detrended fluctuation analysis (DFA), velocity, stride length, and arm swing velocity. Spatiotemporal gait measures and DFA of stride time were compared using repeated measures multivariate analysis of variance.

**Results:**

Analyses included 15 participants with Parkinson disease (mean [SD] age, 69 [6] years; 11 [73%] male; mean [SD] Hoehn and Yahr Parkinson disease stage. 2.3 [0.6]; mean [SD] age of onset, 63 [7] years) and 15 controls (mean [SD] age, 69 [5] years; 11 [73%] male). Stride time DFA was increased during the fractal metronome condition (α = 0.200; SE, 0.024; *P* < .001) vs no cue, the regular metronome (α = 0.320; SE, 0.032; *P* < .001), and music (α = 0.219; SE, 0.030; *P* < .001); worsened with the regular metronome vs no cue (α = −0.120; SE, 0.037; *P* = .003); and was not statistically different during music vs no cue (α = −0.019; SE, 0.031; *P* = .54). Music was associated with increased velocity (mean [SE] change, 0.041 [0.015] m/s; *P* = .01), stride length (mean [SE] change, 0.047 [0.013] m; *P* = .001), and arm swing velocity (mean [SE] change, 27.10 [7.33] °/s; *P* = .001) compared with no cue; velocity (mean [SE] change, 0.030 [0.011] m/s; *P* = .03), stride length (mean [SE] change, 0.034 [0.009] m; *P* = .002), and arm swing velocity (mean [SE] change, 34.7 [8.5] °g/s; *P* = .001) compared with the regular metronome; and increased stride length (mean [SE] change, 0.028 [0.009] m; *P* = .01) and arm swing velocity (mean [SE] change, 37.52 [7.89] °/s; *P* < .001) compared with the fractal metronome. Analyses restricted to participants with Parkinson disease found similar trends, with reduced levels of significance due to the smaller sample.

**Conclusions and Relevance:**

In this case-control study, walking to the fractal metronome was associated with improved stride time fluctuations compared with the regular metronome or music. Walking to music was associated with improved velocity, stride length, and arm swing velocity compared with either metronome condition.

## Key Points

**Question:**

Are auditory cues associated with differences in walking patterns in people with Parkinson disease compared to healthy older adults?

**Findings:**

In this case-control study of 30 older adults, including 15 participants with Parkinson disease and 15 healthy controls, music was associated with faster walking speed, longer steps, and better arm movement, without worsening step timing compared with a standard metronome. Additionally, a fractal metronome was associated with more natural step timing patterns compared with no cue, while a regular metronome was associated with making these patterns worse.

**Meaning:**

These findings suggest that music may be a better auditory cue for improving walking in people with Parkinson disease and in healthy older adults compared with a metronome; future studies should focus on identifying types of music that naturally support healthy rhythm patterns.

## Teaser

This case-control study examines the associations of 3 auditory cues vs no cue with gait measures among older adults with Parkinson disease and healthy controls.

## Introduction

Parkinson disease (PD) is associated with common and disabling gait disturbances, including a forward flexed posture, deceased arm swing, reduced walking velocity, and uneven short steps.^1^ Healthy gait contains natural stride-to-stride fluctuations (variability). In PD, it is well established that gait variability increases, as traditionally measured by SD or coefficient of variation.^2^ When looking at patterns of gait variability over time, healthy gait variability exhibits long-range correlations with a fractal-like structure.^3,4,5^ In aging and PD, this fractal-like structure breaks down, becoming more random resembling white noise, and has been linked to balance dysfunction and falling.^6^ Navigation of natural environments progressively becomes more challenging with age, especially with diseases like PD. Collectively, these impaired gait features have been strongly associated with diminished overall mobility and a worse quality of life.^7,8^ Many, if not most, of these gait features are less responsive or nonresponsive to pharmacotherapy, especially with PD progression.

Cueing has been studied as a therapy to improve impaired gait for more than 75 years^9^ and has been established as an effective intervention for PD, with recommendations for incorporation into rehabilitation programs.^10,11,12^ Rhythmic auditory cues (RAC) with a regular beat have been shown to improve gait velocity and stride length, with mixed results on cadence and traditional variability.^13,14,15,16,17,18^ However, listening to a beat with a fractal structure of variability is a more novel approach that has begun to show promise in restoring healthy gait variability, exhibiting long-range correlations in older adults and PD.^19,20,21^

RACs often come in the form of a metronome or music. Music, in addition to providing rhythm, can evoke emotion, or affect, such as feelings of pleasantness or arousal.^22,23^ Pleasurable responses to music correlate with activity in brain regions associated with reward and emotion, which can lead to dopamine release in the striatal system.^24,25^ Evoking emotion has also been shown to change movement in healthy older adults and those with PD, indicating potential to improve gait beyond that of rhythm alone.^26^ Yet, the optimal RAC for enhancing all gait variables remains unknown.

We aimed to determine whether any of 3 RAC conditions was associated with improved gait performance by comparing no auditory cue with a regular-beat metronome, a fractal-beat metronome, and music. We tested a cohort of participants with PD and compared them with healthy older adult controls to understand whether participants with PD and controls respond differently to auditory cues. Our main hypothesis was that the 3 cues would be associated with unique gait responses due to different beat variability structures and varied modalities of beat delivery. We also hypothesized that participants with PD would be more responsive than controls due to worse gait deficits, as cues are used to bypass faulty networks.

## Methods

This case-control study was approved by the University of Florida institutional review board. All participants provided written informed consent prior to participation. This study is reported following the Consolidated Standards of Reporting Trials (CONSORT) reporting guideline for a randomized crossover study.^27^

A total of 30 participants were recruited, including 15 persons with idiopathic PD diagnosed by a movement disorders neurologist and 15 age-matched (±2 years) controls. Qualified candidates with PD (ie, those with Hoehn and Yahr PD stages 2 to 3) were recruited through the University of Florida Fixel Institute for Neurological Diseases. Control candidates were recruited from the general community. Participants with PD took their parkinsonian medications on arrival to ensure testing was performed during the participant’s optimally medicated state. All participants were required to be ambulatory without the use of an assistive device, aged 45 to 80 years, and able to adhere to trial procedures. Participants were excluded if they had a history of neurological (other than PD), vestibular, unstable orthopedic, or other medical condition that could impair walking function precluding their ability to participate.

Participants visited the Applied Neuromechanics Laboratory at the University of Florida wearing athletic clothing and comfortable walking shoes. Participants completed a medical history form, Physical Activity Readiness Questionnaire, and Falls Efficacy Scale. Participants with PD were evaluated with the Unified Parkinson Disease Rating Scale part III, which was recorded and later rated by a fellowship-trained movement disorders neurologist (L.A.). After measuring height and weight, participants were instrumented with Opal sensors (Clario) containing triaxial accelerometers, gyroscopes, and magnetometers, worn via an elastic strap over the feet, wrists, sternum, and lower back. An access point for wireless data transmission was placed on the perimeter of the walking space to record data. Gait parameters were computed via validated algorithms within the Mobility Lab software.^28,29^

### Protocol

Participants began with a 2-minute walking trial to assess cadence at their self-selected comfortable pace. Thereafter, four 5-minute walk trials under 4 different auditory conditions were performed: no auditory cue, regular metronome, fractal metronome, and music. Mean cueing tempos were adjusted to match each participant’s self-selected cadence, as measured during the initial 2-minute walk. Cues were delivered via an MP3 player and headphones. Conditions were randomized using Research Randomizer software version 4.0 (Urbaniak, G.C. & Plous, S.). On completion of each trial, participants rated their emotional state via an affect grid.^30,31^ Optional rest was provided between conditions.

All walking trials were performed around the perimeter of a gymnasium measuring 29 × 15 m. In the no cue condition, participants were asked to walk at a comfortable pace for 5 minutes. For all RAC conditions, participants were instructed to walk to the beat as best they could. In the regular metronome condition, a regular repeated isochronous tone was played. In the fractal metronome condition, the variability in the length of time between beats exhibited long-range correlations over time to create a fractal-like structure. The magnitude of the time variability between beats was determined by the SD of the participant’s stride time during the initial 2-minute walk. This individualized fractal auditory cue was created by embedding pink noise (1/f noise representing fractal processes) into a series of repeated tones (same tone as regular metronome).^32^ Participants were informed that the beat intervals would be slightly varied. In the music condition, participants listened to “Stayin’ Alive” by the Bee Gees (RSO Records). This music selection was chosen because of its upbeat nature and it has a recorded tempo (103.6 bpm) that is within range of the average cadence for a person with mild- to moderate-stage PD.^33^ The customized music files were created via the Djay 2 application for iPhone (algoriddim, version 2.8.1).

### Data Processing

Walking trials were recorded with the Opal system.^34^ Gait measures were broken down on a stride-by-stride basis when exported to Excel version 2016 (Microsoft). Velocity was defined as mean gait speed, calculated as stride length divided by stride time. Cadence was defined as steps taken per minute. Stride length was defined as the distance between 2 consecutive heel strikes. Stride time was defined as the duration between 2 consecutive heel strikes. Trunk transverse range of motion was defined as the angular range of the thoracic spine in the transverse plane. Arm swing velocity was defined as the peak angular velocity of the arms. Arm range of motion was defined as the norm of the 3-dimensional arm rotation vector during arm swing.

Fractal scaling of the stride time series’, during all 4 auditory conditions, were determined by a detrended fluctuation analysis (DFA) algorithm implemented into MATLAB R2016a (version 9.0; MathWorks).^35^ By using DFA, a resultant scaling exponent (α) is computed, with α of approximately 0.5 indicating unpredictable white noise, while α approximately 1.0 indicated 1/f-like noise and long-range correlations.^36^ Participants were asked to walk for 5-minute time periods to determine the fractal scaling of stride interval time series.^19,37,38^

### Statistical Analysis

To determine whether participants were able to entrain their steps to the tempo of each auditory condition, cadences during regular metronome, fractal metronome, and music conditions were compared with no cue cadence for both groups using paired *t* tests. Gait performance change scores were calculated by subtracting no cue gait values from regular metronome, fractal metronome, and music gait values. A 1-way multivariate analysis of variance (MANOVA) was performed to control familywise error across comparisons of baseline gait characteristics of PD and control groups as determined during the 5-minute no cue walking condition. Correlations among dependent variables were examined to assess multicollinearity. We expect that for gait-related measures, some variables were highly correlated; this pattern supports the use of a multivariate analytic approach rather than separate univariate tests. Homogeneity of covariance matrices was considered in the context of the balanced repeated-measures design. If there are equal observations across conditions and reliance on Pillai trace, the analysis is considered robust to potential violations.

To compare each auditory cue’s associations with gait and to determine whether PD and control groups respond differently to auditory cues, we performed a 2 × 3 mixed-design MANOVA with group as a between-participant factor and cue condition as a within-participants factor. Gait performance change scores in velocity, stride length, arm swing velocity, and stride time DFA among regular metronome, fractal metronome, and music conditions were compared within each population. Change scores in velocity, stride length, arm swing velocity, and stride time DFA were additionally compared with zero via a 1-sample *t* test to determine whether the regular metronome, fractal metronome, and music conditions were associated with statistically significant changes in gait performance vs no cue. We repeated this analysis in a 1 × 3 design focusing on the participants with PD to ensure that the association remained stable while only comparing the participants with PD with their preintervention state.

Levels of significance for analyses were set at α < .05. As 4 dependent variables were chosen for statistical analysis, we used the Bonferroni post hoc correction method to adjust for the familywise error introduced by multiple comparisons. All comparisons were made in a 2-tailed distribution to show difference in either direction. For analyses with multiple comparisons, we used the Bonferroni post hoc correction and reported the corrected *P* value.

Affect grid ratings were assessed for pleasure and arousal scores via two 2 × 4 (group × auditory condition) repeated measure ANOVAs. Total group analyses were performed in 2017. The 1 × 3 repeated measures MANOVA focused on PD was performed in 2026. Analyses were conducted using Python version 3.12 (Python Software Foundation).

## Results

Analyses included 15 participants with PD (mean [SD] age, 69 [6] years; 11 [73%] male; mean [SD] Hoehn and Yahr PD stage. 2.3 [0.6]; mean [SD] age of onset, 63 [7] years) and 15 controls (mean [SD] age, 69 [5] years; 11 [73%] male). Participant demographic information and baseline gait characteristics are summarized in Table 1. Baseline comparisons of walking performance between individuals with PD and controls were performed from data obtained during the no cue walking condition. Individuals with PD walked significantly slower (mean [SD] velocity, 1.07 [0.22] m/s vs 1.22 [0.13] m/s; *P* = .03) with significantly shorter strides (mean [SD] stride length, 1.15 [0.21] m vs 1.30 [0.11] m; *P* = .02) and significantly reduced arm swing velocity (mean [SD] arm swing velocity, 182 [55] °/s vs 237 [65] °/s; *P* = .02). While DFA values were approximately 8% lower in individuals with PD, this difference did not reach statistical significance. The PD group had a significantly higher fear of falling during daily activities of living as determined by the Falls Efficacy Scale (mean [SD] score, 20.0 [16.5] vs 10.5 [1.1]; *P* = .03). No significant differences in cadence were found between groups or between auditory cueing conditions compared with the no cue baseline, indicating all participants were able to entrain to the beat.

**Table 1.** Participant Demographics and Baseline Gait Characteristics

Variable	Mean (SD)		*P* value
	Parkinson disease	Controls	
Total, No.	15	15	NA
Sex, No. (%)			
Female	4 (27)	4 (27)	NA
Male	11 (73)	11 (73)	
Age, y	69 (6)	69 (5)	.87
Height, cm	173 (9)	175 (9)	.67
Weight, lbs	183 (25)	183 (36)	.96
Age of PD onset	63 (7)	NA	NA
UPDRS-III			
Overall	24.4 (11.4)	NA	NA
Posture	1.0 (0.8)	NA	NA
Gait	0.6 (0.8)	NA	NA
Postural stability	1.0 (1.2)	NA	NA
Schwab and England	85.3 (10.6)	NA	NA
Hoehn and Yahr stage	2.3 (0.6)	NA	NA
Velocity, m/s	1.07 (0.22)	1.22 (0.13)	.03
Cadence, steps/min	112 (10)	113 (8)	.61
Stride length, m	1.15 (0.21)	1.30 (0.11)	.02
Stride time, s	1.09 (0.11)	1.06 (0.07)	.53
Trunk transverse ROM, °	9.03 (2.33)	9.72 (2.75)	.47
Arm swing velocity, °/s	182 (55)	237 (65)	.02
Arm swing ROM, °	43.0 (19.9)	56.9 (17.6)	.05
DFA of stride time, α	0.76 (0.09)	0.82 (0.08)	.07
Falls Efficacy scale	20.0 (16.5)	10.5 (1.1)	.03

Abbreviations: DFA, detrended fluctuation analysis; NA, not applicable; ROM, range of motion; UPDRS-III, Unified Parkinson Disease Rating scale.

**Figure 1.**
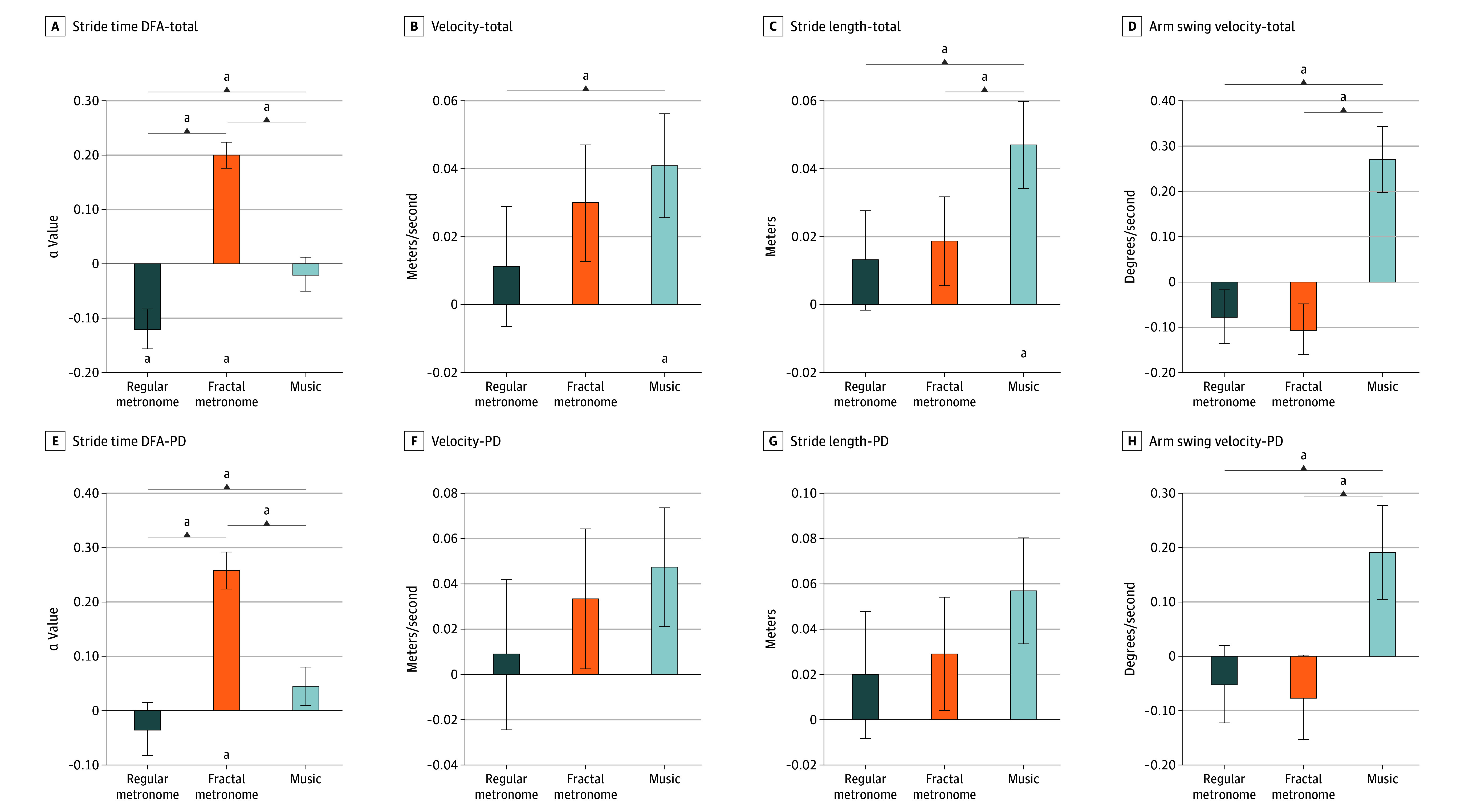
Bar Charts of Change in Gait Measures During Regular Metronome, Fractal Metronome, and Music Conditions Compared With Walking With No Cue PD indicates Parkinson disease. ^a^Statistical significance from baseline. ^b^Statistical significance between conditions (*P* ≤ .0125).

Assumptions for the 2 to 3 mixed-design MANOVA were examined prior to analysis. Although some dependent variables showed modest deviations from normality, the analysis was considered robust, given the balanced within-participant design and the a priori use of Pillai trace, which is resilient to violations of normality and covariance assumptions. Sphericity was assessed only for follow-up univariate tests, with corrections applied as needed. Associations among gait-related outcomes supported a multivariate approach, and potential covariance heterogeneity was mitigated by the balanced design and choice of test statistic. The multivariate analysis failed to identify a significant group-level association; however there was a significant condition-level association (*P* = .002, Pillai trace = 0.65; F_8,80_ = 4.76; *P* < .001). The interaction term failed to reach statistical significance. At the multivariate level of within-participant outcomes, gait responses to auditory cues between PD and HC groups were not statistically different. Significant differences in gait responses were found between among cuing conditions. Total group gait performance change scores were calculated for all auditory cueing conditions from baseline no cue (Table 2). The regular metronome condition was associated with a significant decrease in stride time DFA value (α = −0.120; SE, 0.037; *P* = .003) from zero, whereas the fractal metronome condition was associated with a significant increase in stride time DFA value (α = 0.200; SE, 0.024; *P* < .001) compared with no cue, and the music condition was not associated with a statistically significant change (α = −0.019; SE, 0.031; *P* = .54) (Figure 1). The music condition was associated with significant increases in velocity (mean [SE] change, 0.041 [0.015] m/s; *P* = .01), stride length (mean [SE] change, 0.047 [0.013] m; *P* = .001), and arm swing velocity (mean [SE] change, 27.10 [7.33] °/s; *P* = .001) compared with no cue (Figure 1). For the regular metronome condition, there were no significant differences in velocity (mean [SE] change, 0.011 [0.017] m/s; *P* = .53), stride length (mean [SE] change, 0.013 [0.014] m; *P* = .38), and arm swing velocity (mean [SE] change, −7.65 [5.91] °/s, *P* = .21) compared with the no cue condition. There were also no significant differences in the fractal metronome condition for velocity (mean [SE] change, 0.030 [0.017] m/s; *P* = .09), stride length (mean [SE] change, 0.019 [0.013] m; *P* = .17), or arm swing velocity (mean [SE] change, −10.40 [5.56] °/s; *P* = .07).

**Table 2.** Total and PD Group Condition Gait Change Scores From Baseline

Measure	Condition, mean (SE)		
	Metronome		Music
	Regular	Fractal	
**Total (n = 30)**			
Velocity, m/s	0.011 (0.017)	0.030 (0.017)	0.041 (0.015)^a^
Stride length, m	0.013 (0.014)	0.019 (0.013)	0.047 (0.013)^a^
Arm swing velocity, °/s	−7.65 (5.91)	−10.40 (5.56)	27.10 (7.33)^a^
DFA of stride time, α	−0.120 (0.037)^a^	0.200 (0.024)^a^	−0.019 (0.031)
**PD only (n = 15)**			
Velocity, m/s	0.009 (0.033)	0.033 (0.031)	0.047 (0.026)
Stride length, m	0.020 (0.028)	0.029 (0.025)	0.057 (0.023)
Arm swing velocity, °/s	−5.13 (7.12)	−7.53 (7.75)	19.10 (8.62)
DFA of stride time, α	−0.034 (0.049)	0.260 (0.033)^a^	0.045 (0.035)

Abbreviations: DFA, detrended fluctuation analysis; PD, Parkinson disease.

^a^ Bonferroni-corrected statistical significance from baseline (no cue): *P* ≤ .0125.

At the univariate level, significant associations were observed for the change in velocity (F_2,28_ = 4.17; *P* = .009), stride length (F_2,28_ = 39.45; *P* = .001), arm swing velocity (F_2,28_ = 12.01; *P* < .001), and stride time DFA (F_2,28_ = 2.87; *P* = .002). The music condition was associated with significantly increased velocity (mean [SE] change, 0.030 [0.001] m/s; *P* = .03), stride length (mean [SE] change, 0.034 [0.009] m; *P* = .002), arm swing velocity (mean [SE] change, 34.7 [8.5] °/s; *P* = .001), and stride time DFA (α = 1.01 SE, 0.029; *P* = .005) compared with the regular metronome and significantly increased stride length (mean [SE] change, 0.028 [0.009] m) and arm swing velocity (mean [SE] change, 37.52 [7.89] °/s; *P* < .001) compared with the fractal metronome (Figure 1). The fractal metronome condition was associated with significantly increased stride time DFA compared with the regular metronome condition (α = 0.320; SE, 0.032; *P* < .001) and the music condition (α = 0.219; SE, 0.030; *P* < .001) (Figure 1).

Analyses evaluated the differences in group (PD vs HC), auditory cuing rhythm (regular metronome vs fractal metronome), and their interaction. The repeated-measures multivariate analysis identified a significant group association (F_4,25_ = 2.570; *P* = .03) and a significant condition association (F_8,21_ = 17.564; *P* < .001). The interaction term failed to reach significance. At the univariate level, a group-level association was observed for the change in stride time DFA (mean [SE] difference, 0.139 [0.045]; *P* = .005). During the regular metronome condition, both groups had decreases in DFA values, more in controls than in PD, but the difference was not statistically significant. During the fractal metronome condition, both groups exhibited increased DFA values, with larger changes observed in the PD group. The net change was larger and more positive in PD and smaller in magnitude and negative in controls. A condition-level association was observed for DFA (F_2,28_ = 39.45; *P* < .001). At the univariate level, condition-level associations were not observed for the change in velocity, stride length, or arm swing velocity (Figure 1).

Post hoc analyses in the PD group found similar trends (Figure 1). Fractal metronome cueing was associated with significantly improved stride time DFA compared with no cue (α = 0.257; SE, 0.033; *P* < .001), regular metronome (α = 0.291; SE, 0.041; *P* < .001), and music (α = 0.212; SE, 0.033; *P* < .001). Music cueing also was associated with improved DFA more than regular metronome (α = 0.079; SE, 0.026; *P* = .04). For arm swing velocity, music cueing was associated with significantly increased movement amplitude compared with regular metronome (mean [SE] difference, 24.2 [6.3] °/s; *P* = .005) and fractal metronome (mean [SE] difference, 26.6 [6.6] °/s; *P* = .004), while no significant differences were observed between the metronome conditions. No significant pairwise differences were observed for velocity or stride length between conditions after Bonferroni correction.

**Figure 2.**
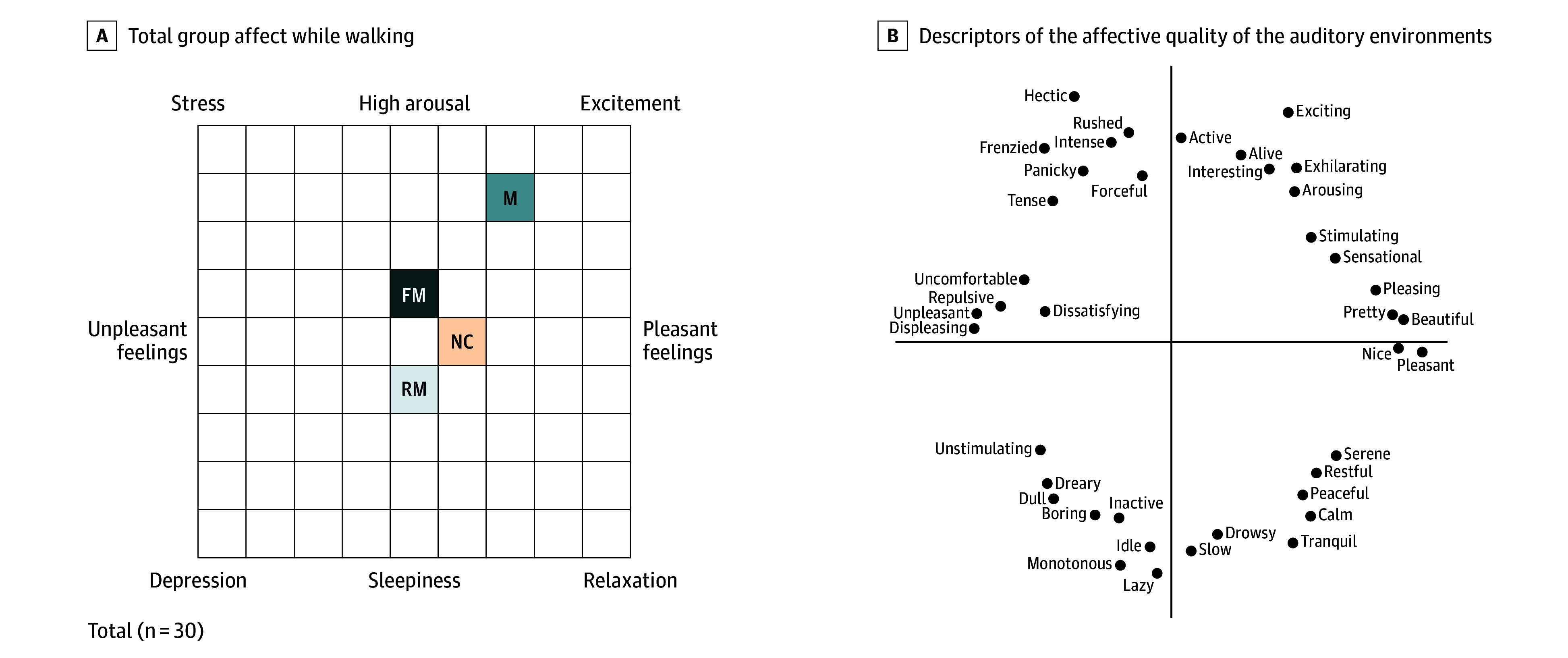
Affect Grid of Emotional State During All Auditory Conditions FM indicates fractal metronome; M, music; NC, no cue; RM, regular metronome; M, music.

**Figure 3.**
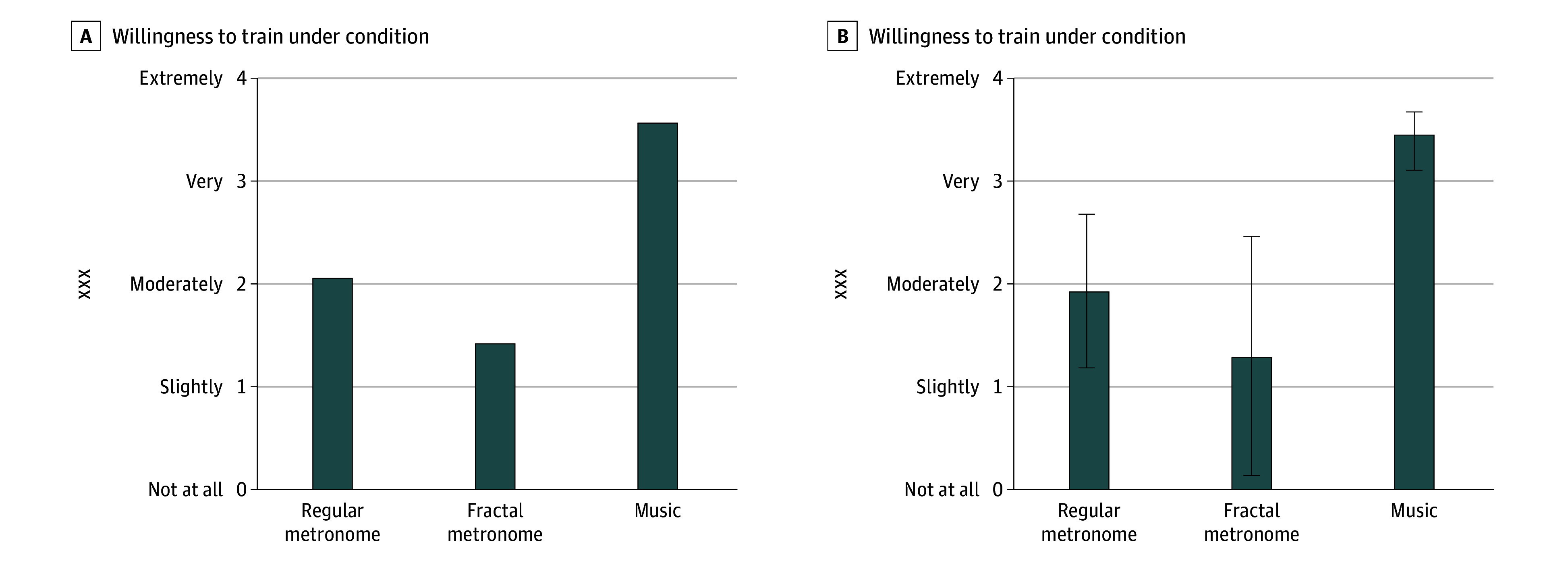
Bar Charts of Willingness to Train Under All Auditory Cueing Conditions

When using the grid, the music condition elicited high ratings in both pleasantness and arousal as compared to the relatively neutral ratings in both categories during the metronome conditions (Figure 2). Participants favored training under the music condition over use of a metronome (Figure 3).

## Discussion

The results of this case-control study suggest that healthy controls and participants with PD successfully adapted their walking to auditory cues and their adaptations were statistically similar. This suggests that the pathways involved with auditory cueing responses may still be intact in PD pathology in mild- to moderate-stage disease, posing a potential target for gait rehabilitation. The regular metronome condition was associated with significantly reducing the healthy structure of stride time variability, whereas fractal metronome was associated with enhancing the healthy structure. Interestingly, music was associated with better walking performance vs a metronome with the same beat. When practitioners use auditory cueing, it is important to appreciate that use of a regular metronome may worsen gait variability. Our data suggest that a fractal metronome may be an alternative option.

Perhaps the most interesting finding of this study was that gait velocity, stride length, arm swing velocity, and stride time DFA were all significantly increased with music compared with regular metronome. Furthermore, walking to music was associated with significant increases in gait velocity, stride length, arm swing velocity, and stride time DFA vs no cue. These data suggest that music was associated with gait improvements beyond what was observed using simple auditory rhythmic stimuli.

There were likely elements present in the music, beyond rhythm, that further enhanced the motor response. Rhythm influences compensatory neural circuits to match a pace-oriented goal and to entrain movement to a set beat.^39^ The amplitude of rhythm, or beat strength, may also impact gait. High-groove music likely elicits enhanced gait synchronization and faster velocity.^40^ Groove is the experience of wanting to move when hearing music.^41^ Our selection of a Bee Gees tune was deliberate as we were interested in the possibility of positive changes in synchronization and velocity.

Music evokes emotion, impacting affect. To assess the emotional impact of the music, along with that of the metronome, we used an affect grid,^30^ a 1-dimensional tool used in psychology to measure and to visualize an individual’s emotional state. The positive emotional response elicited by our musical selection may have contributed to the improved gait parameters.^26^ While the scaling exponent of the “Stayin’ Alive” recording was not able to be determined, it is probable that the value was more fractal in nature compared with a regular metronome.

### Limitations

There were several limitations in this study. Our conclusions were based primarily on the total group rather than participants with PD only. The duration of the walking trials was limited to 5 minutes to prevent fatigue, given the number of conditions tested. Future studies should assess fractal scaling measures over a longer time series. Walking was performed around the perimeter of an indoor gymnasium in a repeated counterclockwise pattern. This could have biased measurements. A treadmill was deliberately not used for this study to avoid eliciting a sensory cue. This study included participants with all PD subtypes and healthy controls. As such, this broader inclusion could have impacted outcomes. Furthermore, translation of the results is limited to the selected Bee Gees song.

## Conclusions

This case-control study found that the use of a fractal metronome was associated with acutely reinstated long-range correlations in stride time fluctuations. Use of a regular metronome was associated with worsened gait scaling. Music, as an auditory cueing modality, was superior to a regular metronome.

## Notes

Supplement.
Data Sharing Statement
