# Supplementary material for: Regular Metronome, Fractal Metronome, and Music for Parkinson Gait
Source: JAMA Netw Open. 2026 Apr 16;9(4):e262744. doi: 10.1001/jamanetworkopen.2026.2744 (PMC13087817; doi:10.1001/jamanetworkopen.2026.2744)
Supplement: Supplement. — Data Sharing Statement [file jamanetwopen-e262744-s001.pdf]

Data Sharing Statement

Sowalsky. Regular Metronome, Fractal Metronome, and Music for Parkinson Gait. *JAMA Netw Open*. Published March 20, 2026. doi:10.1001/jamanetworkopen.2026.2744

Data
Data available: Yes
Data types: Deidentified participant data, Other (please specify)
Additional Information: Open to data sharing with a written request that is reviewed by a data review panel.
How to access data: Please send request to corresponding author cjhass@aa.ufl.edu
When available: With publication

Supporting Documents
Document types: None

Additional Information
Who can access the data: Open to data sharing with a written request that is reviewed by a data review panel.
Types of analyses: Open to data sharing with a written request that is reviewed by a data review panel.
Mechanisms of data availability: After review of a proposal and signed data access agreement.
